# Unusual Loop-Sequence Flexibility of the Proximal RNA Replication Element in EMCV

**DOI:** 10.1371/journal.pone.0024818

**Published:** 2011-09-14

**Authors:** Jan Zoll, Marc M. Hahn, Paul Gielen, Hans A. Heus, Willem J. G. Melchers, Frank J. M. van Kuppeveld

**Affiliations:** 1 Department of Medical Microbiology, Radboud University Nijmegen Medical Centre, Nijmegen Centre for Molecular Life Sciences, Nijmegen, The Netherlands; 2 Laboratory of Biophysical Chemistry, Institute for Molecules and Materials, Radboud University Nijmegen, Nijmegen, The Netherlands; University of Cambridge, United Kingdom

## Abstract

Picornaviruses contain stable RNA structures at the 5′ and 3′ ends of the RNA genome, OriL and OriR involved in viral RNA replication. The OriL RNA element found at the 5′ end of the enterovirus genome folds into a cloverleaf-like configuration. In vivo SELEX experiments revealed that functioning of the poliovirus cloverleaf depends on a specific structure in this RNA element. Little is known about the OriL of cardioviruses. Here, we investigated structural aspects and requirements of the apical loop of proximal stem-loop SL-A of mengovirus, a strain of EMCV. Using NMR spectroscopy, we showed that the mengovirus SL-A apical loop consists of an octaloop. In vivo SELEX experiments demonstrated that a large number of random sequences are tolerated in the apical octaloop that support virus replication. Mutants in which the SL-A loop size and the length of the upper part of the stem were varied showed that both stem-length and stability of the octaloop are important determinants for viral RNA replication and virus reproduction. Together, these data show that stem-loop A plays an important role in virus replication. The high degree of sequence flexibility and the lack of selective pressure on the octaloop argue against a role in sequence specific RNA-protein or RNA-RNA interactions in which octaloop nucleotides are involved.

## Introduction

Cardiovirus, a genus of the family of *Picornaviridae*, is a group of small, nonenveloped RNA viruses. The genus cardiovirus includes two species, encephalomyocarditis virus (EMCV) and Theilovirus, comprising Theiler's murine encephalitis virus (TMEV), Theiler's virus of rats, and Saffold virus (SafV), a human pathogen that is closely related to TMEV [1]. Cardioviruses contain a single-stranded RNA genome of positive polarity of about 7500 nucleotides. The genomic RNA contains a single large open reading frame, preceded by a long 5′-untranslated region (5′ UTR) of approximately 750 nucleotides and followed by a short 3′ UTR of approximately 125 nucleotides. The RNA is translated into a single large polyprotein, which is processed by a virus-encoded protease to yield the individual structural capsid proteins and the nonstructural P2 and P3 region proteins, including the viral encoded RNA-dependent RNA polymerase (RdRp) 3D^pol^ and some relatively stable processing intermediates performing activities in viral RNA replication distinct from their final cleavage products [2]. A small viral peptide (VPg) is covalently attached via a tyrosine residue to the 5′-side of both the plus and minus strand viral RNA.

The cardiovirus infectious cycle involves a number of distinct steps. Following cell entry and virion uncoating, the monocistronic viral RNA acts as an mRNA, directing the synthesis of the large polyprotein. Proteolytic processing of the polyprotein leads to the liberation and activation of the viral proteins involved in viral RNA replication. Replication of the viral plus-strand RNA starts with the synthesis of a complementary minus-strand RNA in the cytoplasm by the RdRp 3D^pol^. Minus-strand RNA serves as a template for new plus-strand RNA molecules. RNA synthesis takes place in the replication complex at the outer surface of virus-induced membrane vesicles [3]. Newly synthesized RNA that is released from the replication complex may go into another round of translation and replication, or be packaged into capsid proteins to produce infectious virus particles. Virus-induced lysis of the host cell accounts for the release of virus progeny.

Replication of the viral RNA depends on the assistance of other viral and host proteins. Initiation of a minus-strand synthesis demands positioning of the RdRp in close proximity to the 3′-end of the plus-strand, whereas the enzyme should be placed at the 3′-end of a minus-strand to initiate a progeny plus-strand. Since the 3′-ends of the complementary strands are dissimilar, the replication machinery recognizes two different types of origins of replication to initiate synthesis of plus and minus-strands, referred to as oriL and oriR, respectively [4], [5].

In enteroviruses, OriL is well defined and is a multifunctional element involved in both viral genome replication and translation [6]. This element folds into a so-called cloverleaf structure and interacts with diverse viral and host proteins [4]. The oriL contains regions with functional domains for both minus and plus strand RNA synthesis and ensures proper orientation of proteins relative to each other and to the RNA template [7]. The existence and functional importance of a ribonucleoprotein complex involving oriL interacting with the virus protein 3CD and the cellular factor PCBP (poly C binding protein) has been described [8]. Formation of this RNP complex might stabilize the OriL RNA structure [9]. The RNP complex mediates the synthesis of a new plus-strand RNA using the minus strand as a template. In contrast to the enterovirus 5′UTR, RNAs from other picornavirus genera do not harbor a cloverleaf structure. A 5′ proximal single large stem-loop structure is found in the genomes of cardioviruses, aphthoviruses, parechoviruses, and kobuvirus [10]. Kobuvirus 5′ proximal RNA element was shown to interact with viral proteins from the P3 region, implicating a role in viral RNA replication [10]. Aphthovirus genomes harbor a large 360 nucleotide stem-loop structure at its 5′end. This so-called S-region was shown to interact with the 3′UTR [11]. The 5′ large stem-loop of EMCV is followed immediately by two pseudoknots structures [12]. Similar RNA structures have been described for parechovirus [10], [13]. Little is known about the function of the 5′ proximal stem-loops in virus replication. However, sequences of this region are phylogenetic well conserved within species of the various genera, suggesting a crucial role of the RNA elements in viral life cycle. In this study, we explored the structure of the SL-A apex by NMR spectroscopy and the structural aspects by studying the effect of engineered mutations on mengovirus replication.

## Results

### The apical loop of EMCV SL-A is an octaloop

**Figure 1:**
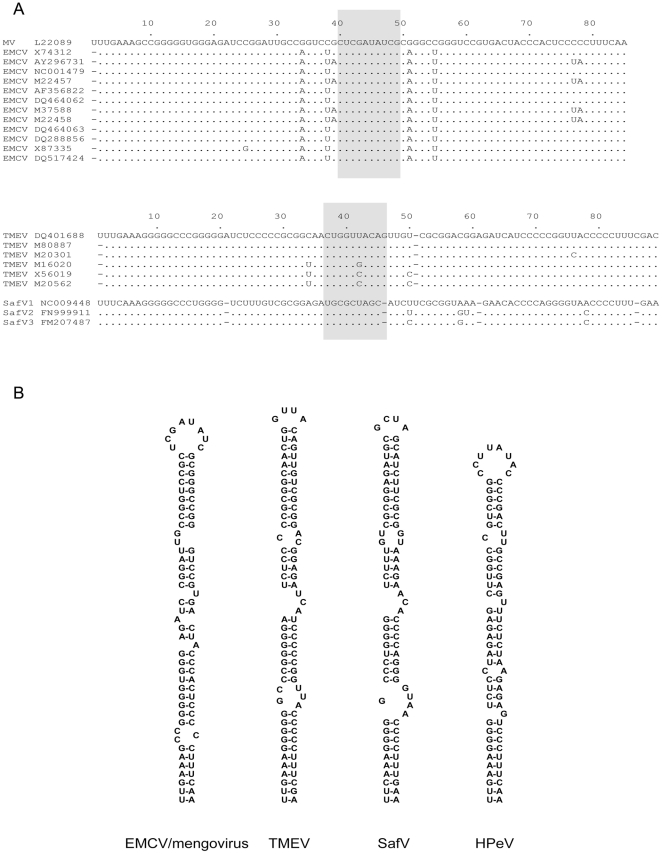
Conservation of cardiovirus OriL SL-A element. (A) Multiple alignment of SL-A of the cardiovirus OriL. The upper part shows the alignment of the EMCV subgroup of the cardiovirus genus. The lower part shows the alignment of the theiloviruses, including the human Theiler's like virus. The apical part of the stem-loop is shaded and includes the EMCV SL-A octaloop and the Theilovirus SL-A tetraloop with two closing base-pairs. (B) Secondary structures of OriL SL-A elements from cardioviruses and parechovirus 5. Structures were calculated using MFold [14].

As a first approach to gain more insight into the secondary structure of the cardiovirus 5′UTR SLA, a multiple alignment was made of available RNA sequences in combination with in silico folding using MFold [14] (Figure 1). The primary structure of the cardiovirus SL-A region appeared to be phylogenetically conserved only at species level (Figure 1A). However, folding of SL-A showed a high degree of similarity in the secondary structures of this element within the entire cardiovirus genus. SL-A is formed by the proximal 85 or 86 nucleotides of the viral genomic RNA (Figure 1B). Apart from the composition and positions of the interhelical bulges, a striking difference was observed in the composition of the apical loop of the EMCV/mengovirus and Theiloviruses: the apical loop of EMCV/mengovirus SL-A consists of an octaloop closed by a C-G base pair, while the Theilovirus SLA apical loop contains a GCUA or GCUU tetraloop closed by a C-G, G-C, or U-A base pair.

### Solution structure of mengovirus SL-A apical loop

Tertiary structures of stable GCUA and GCUU tetraloops have been described previously [15]. The octaloop found in EMCV SL-A consists of a highly conserved UCGAUAUC sequence and might be folded into a stable GAUA tetraloop closed by UC/UC tandem pyrimidine base pairs. Tandem noncanonical pyrimidine base pairs have been found previously in the Y-stem of the poliovirus 3′UTR [16], [17]. In that context, the pyrimidine base pairs were stabilized by Watson-Crick base pairs on either side of the pyrimidine base pairs.

**Figure 2:**
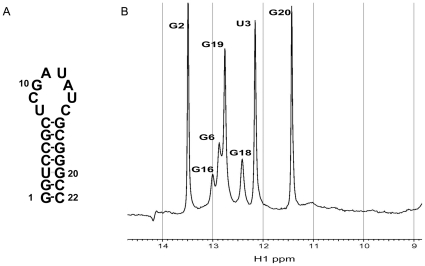
Iminoproton assignment in the apical part of SL-A of the cardiovirus oriL. (A) Secondary structure of the RNA element used for NMR studies. (B) 1D iminoproton spectrum (600 MHz) recorded in H_2_O/10% D_2_O. Iminoproton assignments are indicated.

In order to determine the possible formation of GAUA tetraloop and CU/UC base pairs, NMR experiments were performed on a short RNA hairpin comprising the upper 22 nucleotides of the mengovirus SL-A (Figure 2A). Figure 2B shows the 1D iminoproton spectrum. Iminoproton resonances could be assigned by 2D NOESY experiments in H_2_O/D_2_O. Iminoproton resonances were observed for G and U residues forming base pairs within the stem, including the U3-G20 non-Watson-Crick base pair. Iminoproton resonances could be assigned neither to U residues in the proposed CU/UC base pairs nor to the G residue of the potential GAUA tetraloop. So, these experiments demonstrate that the apical loop of EMCV SL-A consists of an unstructured octaloop.

### Random mutations are well tolerated in the SL-A apical loop

**Figure 3:**
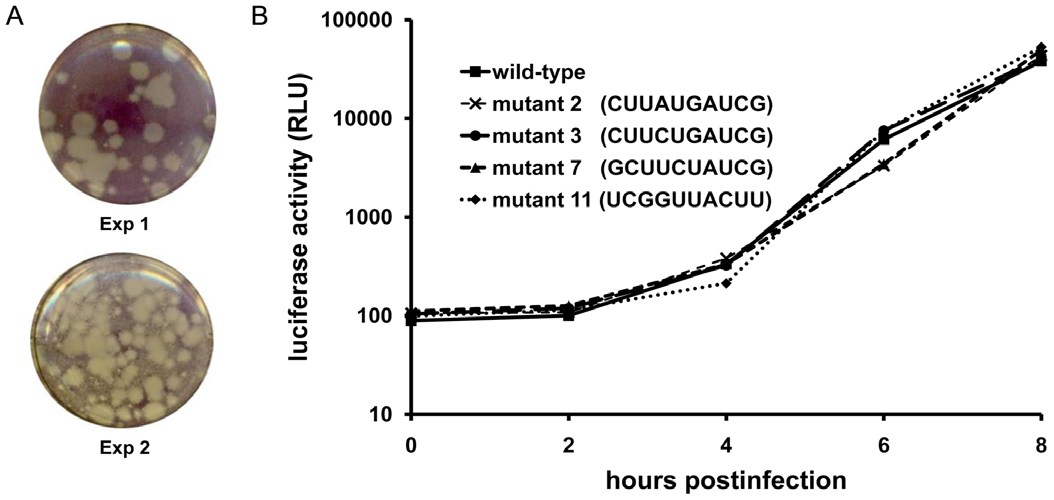
In vivo SELEX of mengovirus OriL SL-A. (A) Plaque assay. L929 cells were infected with a pool of virus containing randomized SL-A. Cells were stained with MTT as described in the text. Two wells from two independent plaque assays are shown (Exp 1 and Exp 2). Individual plaques were picked and further analyzed. (B) Luciferase activity produced by wild-type and four mutants obtained from the in vivo SELEX experiments. Mengovirus OriL SL-A mutations were introduced into the mengovirus luciferase replicon pRzpM-Luz. At various time points after infection luciferase was measured.

Previously, in vivo SELEX experiments were performed on the stem-loop D (SLD) of the poliovirus 5′ cloverleaf and showed a clear correlation between structure of the apical SL-D stem-loop and binding of the viral RdRp [15]. Little is known about the role of the 5′ SL-A of EMCV in virus replication. The observed phylogenetic conservation of the RNA element suggests a possible role in RNA replication. To gain insight into the structure-function relationship of SL-A in virus replication, an in vivo SELEX experiment was set up. To this end, a full genomic PCR amplification was performed on a mengovirus cDNA clone [18] using a forward oligonucleotide primer which contained a T7 RNA polymerase promoter region followed by the first 60 nucleotides of SLA in which the octaloop sequence and the closing base pair were randomized. RNA was transcribed in vitro from the resulting PCR product and transfected into L929 cells. Eighteen hours after transfection, cells were frozen and thawed three times. Cellular lysates were used to perform plaque assays on fresh L929 monolayers. Three days after infection, mutants were obtained by picking non-overlapping plaques. Plaque sizes varied from 0.2 mm to 3 mm, similar as observed for wild-type virus plaques (Figure 3A).

Viruses were grown and further characterized by sequence analysis of the 5′UTR and the protein 3CD encoding region. In total, 218 individual plaques were analyzed whereby 18 different variants, including the wild-type sequence, were found (Table 1). Except for the randomized part of SL-A, no additional mutations were found in the 5′UTR and the 3CD encoding region. To rule out possible effects of other compensatory mutations outside SL-A and 3CD, we introduced all observed SL-A mutant sequences into the infectious mengovirus cDNA clone as well as in a mengovirus replicon in which the P1 encoding region is replaced by firefly luciferase [19]. To study virus replication in a single cycle of replication, in vitro transcribed mengovirus replicon RNA was used for the transfection of L929 cells. At various time points after transfection, luciferase activity was measured. Figure 3B shows the results of the wild-type and four different SL-A mutants. RNA replication kinetics of all mutants was similar to wild-type (Figure 3B; data not shown). In order to study the effects of the mutations in multiple rounds of virus replication and virus spread, in vitro transcribed RNA from the infectious cDNA clone was used for the transfection of L929 cells. In all cases, full cytopathic effect was visible 18 hours after transfection of RNA transcribed from the infectious cDNA clone. Viruses obtained after transfection were analyzed by plaque assay. All SL-A mutants showed wild-type like plaque sizes (data not shown). Examination of the mutant sequence data shows that no consensus sequences could be defined. Nucleotide substitutions were observed at all positions within the randomized region. However, no mutants were obtained in which base pairs were introduced that closed the loop and reduced the loop size to six or four nucleotides. There is a tendency of enrichment of U-residues in the randomized region. In 14 out of the 17 mutants found, more U-residues are present in the 10 nucleotide apical region of SL-A than in the wild-type situation. However, seven mutants were obtained in which the closing CG base-pair was changed into a GU or UG wobble base-pair. Therefore, the number of U-residues found in the loop region of the various mutants was not significantly altered. In 10 out of 17 mutants the number of loop U-residues was less than or equal to the number of U-residues present in the wild-type octaloop. No correlation could be found between the frequency of the mutations and the composition of the randomized region. Several mutants were obtained in which the closing C-G base pair was changed into non-base pairing variants (Table 1, mutant numbers 7, 11, 13, 14, 15, and 18). These mutants displayed a wild-type like phenotype as well. No mutations were found outside the randomized region during the SELEX procedure. Thus, the mengovirus SL-A proximal loop tolerates random mutations, yielding unstructured octaloops or even decaloops, without affecting in vitro virus replication.

**Table 1:** In vivo SELEX of mengovirus OriL SL-A.

Mutant nr.	Sequence	Nr. of mutants found
1	CUCGAUAUCG	35
2	CUUAUGAUCG	5
3	CUUCUGAUCG	4
4	UUGAAUUGCG	4
5	UUAAAUUGCG	11
6	UUUAAUUACG	4
7	GCUUCUAUCG	6
8	GUGGUUACUU	28
9	UGAAAUUGCG	4
10	GUCUGUGUUC	5
11	UCGGUUACUU	6
12	UUGAAAUGAG	6
13	AUUUUGGUUG	48
14	CUUCGUUGUU	6
15	UGCAUGUUAU	24
16	GUUAUACACC	5
17	GUUAUCCACU	11
18	UGGUUUUCGC	6

Randomization of 10 nucleotides in the SL-A apical loop. The table shows the mutations found after in vivo SELEX and the number of plaques obtained for each variant.

### Effects of mutations that reduce the size of the loop

**Figure 4:**
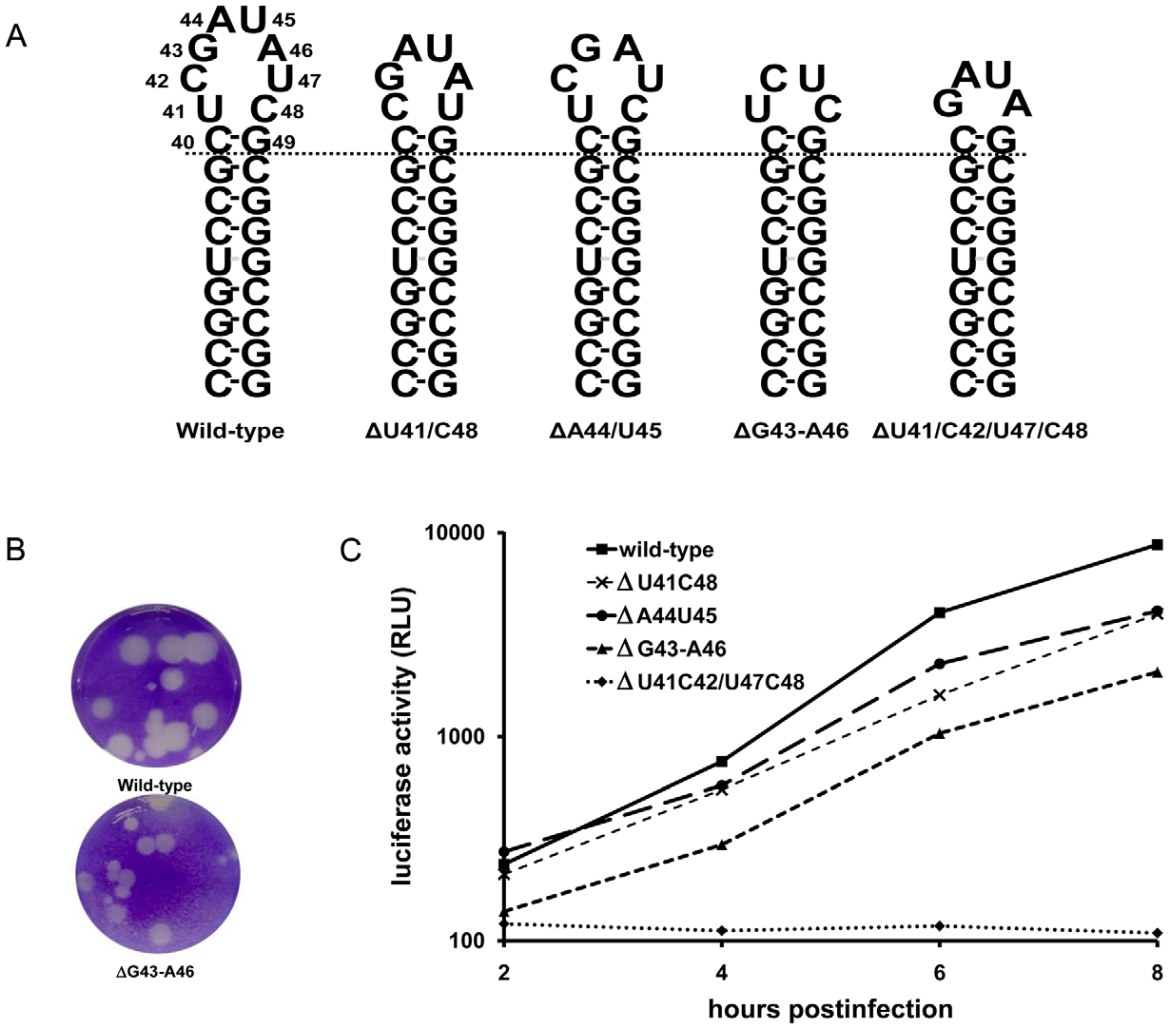
Mutations affecting the mengovirus SL-A loop structure. (A). Mutations introduced by site-directed mutagenesis. (B) Plaque assay of the wild-type mengovirus (up) and the ΔG43-A46 mutant (down). Plaque assay were performed in L929 cells. Cells were stained with crystal violet. (C) Luciferase activity produced by wild-type and the four SL-A loop mutants. Mengovirus OriL SL-A mutations were introduced into the mengovirus luciferase replicon pRzpM-Luz. At various time points after infection luciferase was measured.

To further characterize sequence and structural requirements of SL-A for mengovirus replication, additional mutations were introduced into the mengovirus cDNA (Figure 4, Table 2). First, the requirement for an apical octaloop was examined. Two mutants were made in which two loop residues were deleted resulting in apical hexaloops. Mutations ΔU41/ΔC48 (Figure 4A) and ΔA44/ΔU45 (Figure 4A) were introduced into the infectious mengovirus cDNA clone and the mengovirus replicon. Both mutant RNAs replicated almost as efficient as wild-type viral RNA as was shown in the replicon-luciferase assay (Figure 4C). Consistently, transfection of both in vitro transcribed mutant RNAs gave rise to viruses that displayed intermediate plaque sizes (Table 2, Figures 4B and 4C). Deletions of four residues had a more pronounced effect on virus viability. Mutant ΔG43-A46 (Figure 4A) lacks the central part of the octaloop. Consequently, the apical loop might be folded into a UCUC tetraloop. Virus replication is hampered in this mutant as demonstrated by in the reduction in RNA replication observed in the luciferase assay and the small plaque size phenotype displayed by this mutant virus (Table 2, Figures 4B and 4C). Deletion of the four pyrimidine residues in the octaloop resulted in mutant ΔU41/C42/U47/C48 (Figure 4A). The apical loop of this mutant is expected to fold into a stable GAUA tetraloop with a closing C-G base pair. Using the luciferase assay it was found that the ΔU41/C42/U47/C48 mutations abrogates RNA replication completely (Figure 4C). Consistently, this mutation abrogated virus growth (Table 2). So, efficient virus replication depends on an unstructured SL-A proximal loop of at least six nucleotides. SL-A loops of four nucleotides support replication at a reduced level. In this case, RNA replication depends on the loop constitution: a higher stability causes lower replication.

**Table 2:** Phenotypic characterization of the SL-A loop mutants.

Mutant	Viable virus	Plaque phenotype
Wild-type	+	+++
ΔU41/C48	+	++
ΔA44/U45	+	++
ΔG43-A46	+	+
ΔU41C42/U47C48	−	NA

### Effects of mutations that alter the length of the stem

**Figure 5:**
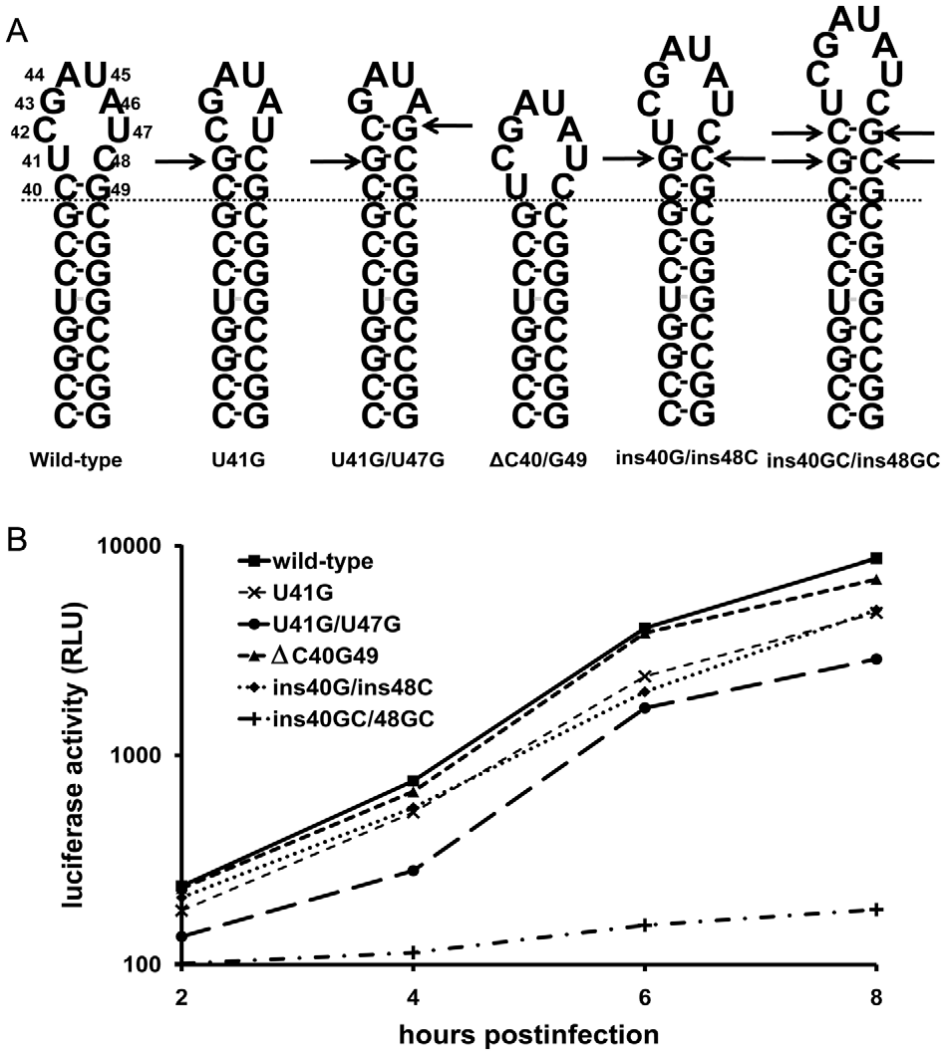
Mutations affecting the mengovirus SL-A stem structure. (A). Mutations introduced by site-directed mutagenesis. (B) Luciferase activity produced by wild-type and the four SL-A loop mutants. Mengovirus OriL SL-A mutations were introduced into the mengovirus luciferase replicon pRzpM-Luz. At various time points after infection luciferase was measured.

Additional mutants were constructed in which the length of the stem was changed. The length of the stem was extended by either replacement of loop residues, thereby creating Watson-Crick base pairs or by inserting extra base pairs into the stem. Mutation U41G (Figure 5A) changed the unpaired U41-C48 into a G41-C48 base pair, thereby extending the stem with one base pair and changing the octaloop into a hexaloop. Transfection of RNA transcribed from the infectious cDNA clone containing the U41G mutation resulted in full cytopathic effect within 18 h after transfection. The U41G virus displayed intermediate plaque size phenotype. RNA replication was at wild-type level as was demonstrated using the luciferase assay (Figure 5B). Mutation U41G/U47G (Figure 5A) changed the unpaired U41-C48 and C42-U47 into G41-C48 and C42-G47 base pairs, thereby extending the stem with two base pairs and changing the octaloop into a GAUA tetraloop. This mutation affected virus replication as was shown by luciferase replicon assay as well as by a reduced plaque size Figures (Table 3, Figure 5B). So, the deleterious effect on virus RNA replication of the deletions in mutant ΔU41/C42/U47/C48, that contains a similar GAUA tetraloop, is partly compensated by extension of the stem with two base-pairs.

**Table 3:** Phenotypic characterization of the SL-A stem mutants.

Mutant	Viable virus	Plaque phenotype
Wild-type	+	+++
U41G	+	++
U41G/U47G	+	+
ΔC40/G49	+	+++
Ins40G/ins48C	+	++
Ins40GC/ins48GC	−	NA

Until now, the length of the stem was modulated by the replacement of loop residues. To examine the effect of stem length without affecting the loop sequence, two additional mutants were made. The length of the SL1 stem was shortened with one base pair in the mutant ΔC40/ΔG49 (Figure 5A). This mutant displayed a large plaque phenotype and a wild-type like RNA replication in the luciferase replicon assay (Table 3, Figure 5B). Mutant ins40GC/ins48GC (Figure 5A) had a stem extended with two base pairs (G-C and C-G). This mutation appeared to be detrimental for virus replication. No cytopathic effect was observed upon transfection and subsequent passaging. No RNA replication was observed in the luciferase replicon assay (Table 3). So, the length of the helical part of the SL-A apex is a determinant for virus replication.

## Discussion

The OriL RNA element is an important structural domain in the picornavirus genome. A stable RNA structure can be found at the 5′ proximal end of the genomic RNA but apart from kobuviruses, aphthoviruses, and enteroviruses, there is hardly any experimental data on the role of the OriL in the replication cycle of other picornaviruses. In this study, we used mengovirus, a strain of EMCV to study sequence and structural requirements of the apical loop of the 5′ stem-loop A of cardiovirus. We describe results of mutational analysis of the mengovirus oriL by in vivo SELEX and site directed mutagenesis.

In previous studies, we and others showed that the binding of enterovirus 3CD to the oriL requires an apical tetraloop in stem-loop D folded in YNMG geometry [15], [20]. In contrast, the apical loop of the EMCV SL-A consists of an octaloop, as shown by NMR in this study, and displays a high degree of sequence flexibility. Despite the very high degree of conservation of SL-A of EMCV, in vivo SELEX revealed that many randomly acquired octaloop sequences are well tolerated and support virus replication at wild-type virus levels. Notably, no variants were obtained in which the apical loop was reduced. In contrast, several mutants were recovered in which the apical loop was enlarged to ten nucleotides.

The results obtained with the mutants in which nucleotides in the apical loop and the upper part of the stem were deleted, inserted or modified, support the hypothesis that EMCV replication requires a unstructured or loosely-structured apical SL-A loop in combination with a stem of defined length. This was shown by the lack of replication of the ΔU41C42/U47C48 mutant which contains a regular RNA stem with a GAUA tetraloop that is further stabilized by the closing C-G base pair [15], [21], [22]. The ΔU41C42/U47C48 mutant did not support viral RNA replication. Deletion of U41 and C48 in mutant ΔU41C48 on the other hand, resulted in viable virus with a wild-type like phenotype. Here, the apical loop consists of a hexaloop, including the GAUA sequence. Stable tetraloop formation is hampered by the lack of a closing base pair. In contrast to mutant ΔU41C42/U47C48, the mutant ΔGAUA(43-46) in which the four central residues of the octaloop are deleted is viable. The detrimental effect of stabilization of the apical loop on RNA replication can partly be rescued by extension of the stem as was observed in mutant U41G/U47G. This mutant contains a SL-A element with similar size as the wild-type stem-loop. Despite the presence of a GAUA tetraloop and a C-G closing base pair, this mutant supported RNA replication. Surprisingly, extension of the stem without changing the octaloop in mutant ins40GC/ins48GC appeared to be detrimental for virus replication. No explanation was found for this result so far.

Previously, Nateri and colleagues [13] showed that replacement of the apical loop of parechovirus SL-A by the EMCV equivalent yielded a virus with wild-type growth characteristics. However, deletion of 8 base pairs from the top of parechovirus SL-A had a detrimental effect on virus viability. These findings show that, similar to EMCV, parechovirus tolerates different octaloop sequences without affecting virus replication in vitro while reducing the length of SL-A stem is detrimental for parechovirus replication [13]. Surprisingly, it seems that SL-A elements cannot be exchanged between cardiovirus genera without consequences. By mutating U41 and U47 into G residues, a tetraloop structure was created that mimicked the Theilovirus apical tetraloop. However, replication was severely diminished in the virus harboring these mutations. Overall, our data suggest that EMCV replication requires an invariable length of SL-A and a certain degree of flexibility of the octaloop as determinants for virus replication. The degree of stability of the octaloop might be a crucial factor for the role of SL-A in virus replication. Studies to the effect of loop size and composition showed that RNA loop stability of loops larger than 5 nucleotides is inversely proportional to loop size rather than loop composition [23], [24].

RNA stem-loop structures can function in a number of ways. Terminal loops and internal bulges can form sites interacting with proteins or other nucleic acid partners. Previously, a number of stem-loop structures containing octaloops were described [25], [26], [27]. Bouvet and colleagues [25] determined the solution structure of a nucleolin-recognition element (NRE) found in pre-rRNA. Although an octaloop was predicted, the NRE appears to contain a seven-nucleotide loop. NMR spectroscopy experiments revealed stacking of a number of A-bases within the loop. However, the overall loop-structure was poorly defined and seems to change between different conformations. Maris and colleagues [26] described the structure of an apoB mRNA stem-loop. This RNA element is the docking site for RNA editing protein complexes, and the apex is formed by a 5′-ACAAUUUG-3′ octaloop closed by a U-A base pair. Although the loop was unstructured and flexible, helical stacking of ACA bases at the 5′-end of the loop was observed. The flexibility of the loop and the upper part of the apoB mRNA stem-loop appeared to be essential for protein binding. A more defined structure of an octaloop was described by Lebars and colleagues [27]. The structure of the bacterial 23S rRNA hairpin-35 stem-loop was dissolved by NMR spectroscopy. This RNA element forms a docking site for rRNA methyltransferase (RlmA^II^). The hairpin contains a 5′-GUUGAAAA-3′ octaloop that is closed by a Watson-Crick C-G base-pair. The loop folds independently of the stem and is stabilized by a sheared G-A base-pair. NMR spectroscopy revealed that the loop structure is well defined and have a stretch of stacked nucleotides formed by the GAAA bases, preceded by two U bases stabilized by stacking between the two G bases. A certain degree of structure formed by non-Watson-Crick base pairs and base-stacking can be recognized in the octaloops described above. Until now, no evidence for interactions between bases in the mengovirus SL-A loop was found. Despite the unstructured and flexible nature of the RNA octaloops in the NRA and apoB mRNA, these elements are involved in specific RNA-protein interactions.

The in vivo SELEX approach was previously used in a study to the structure-function relationship of the stem-loop D apical loop of the poliovirus OriL element, the binding-site for the viral polymerase [15]. A limited number of viable mutants were found displaying a similar folding of the apical tetraloop. Since randomization of the SL-A apical loop results in many mutants without a consensus sequence but displaying wild-type like virus replication makes it unlikely that the mengovirus SL-A octaloop is involved in direct sequence specific RNA-RNA or RNA-protein interactions. Nevertheless, variability of the upper part of SL-A is limited in length and apical loop-size. The reason for this remains to be established.

A similar influence of length of RNA helical parts on virus replication was found for the enterovirus OriR element [28]. Insertion or deletion of an extra base-pair causes a clockwise or counter-clockwise rotation of approximately 33° of the distal portion of the stem [28]. Although no RNA partner is known for the interaction with mengovirus SL-A, it is possible that the reduction of virus replication caused by inserting or deleting base-pairs in the helical part of the SL-A apex is due to a spatial disorientation of the apical loop. For example, the differences in phenotype displayed by U41G/U47G and ins40GC/ins48GC, two mutants with similar stem-lengths, might be caused by a different spatial organization of the apical stem-loop.

It is likely that the constitution of the apical stem of SL-A is a determinant for the formation of tertiary RNA structures, essential for virus replication. Multiple alignment of EMCV SL-A sequences demonstrates a high degree of conservation of both stem and loop of this RNA element. This seems a contradiction with the observed tolerance of random apical loop sequences. However, minor differences in replication efficiency between various sequences supporting wild-type like replication may finally result in the selection of the most optimal constitution found in nature.

## Materials and Methods

### Sequence alignment and prediction of RNA secondary structures

Nucleotide and amino acid sequences were aligned using ClustalW [29]. Prediction of stem-loop structure was performed using the MFOLD 3.2 program [14].

### NMR spectroscopy

The 22-mer RNA oligonucleotide was purchased from ABI GmbH (Göttingen, Germany). The final concentration of the RNA sample was 0.5 mM. The pH was adjusted to 6.8 (meter reading). NMR spectra were acquired on Varian Inova 600 MHz and 800 MHz spectrometers. For the assignment of exchangeable imino protons, two-dimensional NOESY spectra were recorded in 90% H_2_O, 10% D_2_O at 15°C and 800 MHz using a jump return pulse for water suppression [30].

### Randomization of 5′ SL-A apical loop

Mutations were introduced into an infectious mengovirus cDNA by PCR using synthetic oligonucleotides (Sigma-Aldrich, The Netherlands). An oligonucleotide containing 10 random residues in 5′ SL-A apical loop and the closing base pair sequence was used as forward primer. The infectious mengovirus cDNA construct pM16.1 [18] functioned as template in a PCR using Phusion DNA polymerase (Finnzymes, Finland) according to the protocol of the manufacturer.

### Construction of 5′SL-A mutants

The 5′ SL-A mutants were generated by PCR amplification using the infectious mengovirus cDNA clone pM16.1 [18]. The region between the RsrII site (position 34) and the SpeI site (position 2218) was amplified using a forward primer including the SL-A mutation. The amplicon was digested with RsrII and SpeI and ligated into pM16.1 or RzpM-Luz, a mengovirus replicon in which the P1 encoding region is replaced by firefly luciferase [19] digested with the same set of restriction enzymes. Mutations were confirmed by sequence analysis.

### Cells and viruses

Virus propagation and viral RNA transfections were performed with mouse fibroblasts (L929 cells) and HeLa cells. L929 cells and HeLa cells were obtained from ATCC. The cells were grown in minimal essential medium, MEM Glutamax (Invitrogen), supplemented with 10% fetal bovine serum (FBS), 100 U penicillin per ml and 100 mg streptomycin per ml (Sigma).

### RNA transcription and transfection

pM16 and RzpM-Luz cDNAs containing 5′ SL-A mutations were linearized by digestion with BamHI. PCR products comprising the full length mengovirus cDNA including a 5′ T7 RNA polymerase promoter and the randomized 5′ SL-A apical loop or linearized cDNAs served as templates for *in vitro* transcription by T7 RNA polymerase as previously described [31]. L929 cells were transfected in duplicate by a DEAE-dextran method [32], [33] and incubated at 36°C and 5% CO_2_ for eight hours. Then, the cultures were subjected to three cycles of freezing/thawing. Cellular debris was removed by centrifugation for 15 min at 2000× g. Cellular lysates were used for plaque assay.

### Plaque-assay

Six-well dishes with >90% confluent monolayer of L929 cells were incubated with 10-fold virus dilutions in MEM for 1 h and rocked every 15 min. After absorption, the cells were overlaid with 2.5 ml of M199 (Sigma) containing 3% FBS, 1% glutamine, 0.5% gentamicine, 0.5% penicillin, 0.5% Funguzone, 1% MgCl_2_ and 1.3% Na-bicarbonate in 0.6% agar (1∶1 mixture with 2×M199). At 48 h postinfection, cells were either fixed and stained with crystal violet [34] or stained with MTT. Plaques were stained by the addition of 0.1 volume of 5 mg/ml MTT (Sigma) in PBS and 3 hours of incubation at 37°C. Single plaques were picked from MTT stained cultures and purified [34].

### Sequencing of recovered virus RNA

Viral RNA extraction was performed using the GenElute™ Mammalian Total RNA Miniprep kit (Sigma). RNA was reverse transcribed using Super RT (HT Biotechnology) and random hexamer primers. cDNA was PCR-amplified using Phusion DNA polymerase and specific oligonucleotides comprising the genomic region between position 1 and 350. SL-A mutations were confirmed by sequence analysis.

### Luciferase assay

Upon transfection of cells with *in vitro* transcribed RzpM-Luz RNA, translation of the viral RNA gives rise to luciferase activity in the cytoplasm. Replication competent wild-type and mutant viral RNA will produce progeny plus-strand RNA and consequently display an enhanced luciferase activity. L929 cells, grown in 24-well plates to a confluence of 80%, were transfected as described above. At 2, 4, 6, and 8 hours post-transfection cells were lysed in 100 µl Passive lysis buffer (Promega). Luciferase activity was assayed using the Luciferase Assay System (Promega) and quantified using a Promega GloMax luminometer.
